# Microstructure and local electrical behavior in [(Nd_2_Ti_2_O_7_)_4_/(SrTiO_3_)_*n*_]_10_ (*n* = 4–8) superlattices

**DOI:** 10.1039/c8ra00824h

**Published:** 2018-03-21

**Authors:** Thomas Carlier, Anthony Ferri, Sébastien Saitzek, Marielle Huvé, Alexandre Bayart, Antonio Da Costa, Rachel Desfeux, Antonello Tebano

**Affiliations:** Univ. Artois, CNRS, Centrale Lille, ENSCL, Univ. Lille, UMR 8181, Unité de Catalyse et Chimie du Solide (UCCS), Faculté des Sciences Jean Perrin Rue Jean Souvraz SP18 F-62300 Lens France rachel.desfeux@univ-artois.fr +33 321791771; CNR-SPIN, Department of Civil Engineering and Computer Science Engineering, University of Rome Tor Vergata, Via del Politecnico 00133 Rome Italy

## Abstract

Artificial [(Nd_2_Ti_2_O_7_)_4_/(SrTiO_3_)_*n*_]_10_ superlattices (*n* = 4 and 8) were successfully epitaxially grown on SrTiO_3_ substrates by pulsed laser deposition using the *in situ* high energy electron diffraction reflection diagnostic. The crystallographic relationships between Nd_2_Ti_2_O_7_ (NTO) and SrTiO_3_ (STO) (layers and substrate) were: [100]_NTO_//[001]_STO_, [010]_NTO_//[1̄10]_STO_, and (00*l*)_NTO_//(110)_STO_. Nanoscale current variation was detected on both superlattices, with the (NTO_4_/STO_4_)_10_ heterostructure showing a higher density. The (NTO_4_/STO_4_)_10_ sample did not show a piezoelectric response when measured by piezo-force microscopy (PFM), while ambiguous piezoactivity was observed on the (NTO_4_/STO_8_)_10_ superlattice. Scanning transmission electron microscopy energy dispersive spectroscopy analysis showed the diffusion of Nd^3+^ cations on Sr^2+^ sites in SrTiO_3_ structure into the multilayers, which was more pronounced when the value of *n* was lower. These particular nanoscale electrical behaviors, evidenced by electrical conducting channels and misleading PFM signals, were mainly attributed to the presence of oxygen vacancies in the SrTiO_3_ layers at higher concentrations near the interface and to the mixed valence state of the titanium (Ti^3+^/Ti^4+^). This work showed the strong influence of interface structure on nanoscale electrical phenomena in complex oxide superlattices.

## Abstract 2

Artificial [(Nd_2_Ti_2_O_7_)_4_/(SrTiO_3_)_*n*_]_10_ superlattices were epitaxially grown. Local conductivity and misleading PFM signals were mainly attributed to the oxygen vacancies in the SrTiO_3_ layers and to the mixed valence state of the titanium.

## Graphical abstract

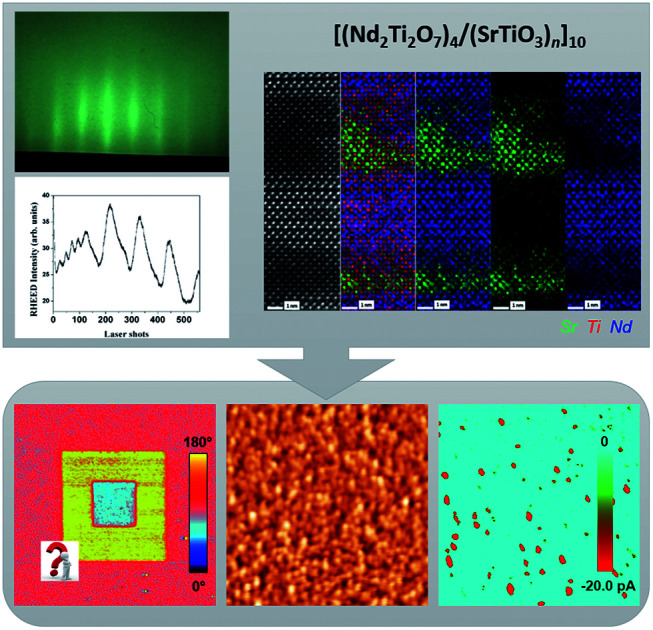

## Introduction

With the development of nanotechnology, transition metal oxide thin films with perovskite-type structures have been increasingly investigated in recent years due to their recognized technologically important properties, such as high-temperature superconductivity, colossal magnetoresistance, ferroelectricity and, more recently, multiferroicity.^1^ Particular interest has been paid to ferroelectric/piezoelectric lanthanide titanium oxides with a perovskite-layered structure (Ln_2_Ti_2_O_7_ family) due to their high Curie temperatures (*T*_c_, ∼1500 °C) which show promise for applications under harsh environments, such as in transducers for motors and aerospace.^2–4^ These oxides include La_2_Ti_2_O_7_ (LTO), Ce_2_Ti_2_O_7_, Pr_2_Ti_2_O_7_, and Nd_2_Ti_2_O_7_ (NTO) thin films, in addition to Sm_2_Ti_2_O_7_ and Gd_2_Ti_2_O_7_ thin film-stabilized metastable phases.^5–12^ Furthermore, these compounds were lead-free, which is crucial for integrating substances into (nano-)devices because this is now recognized as an important criterion for both the environment and human health.^13^ In contrast, these titanates have been shown to possess many other remarkable properties, including high dielectric constants, nonlinear optical activity, photocatalysis, and photoluminescence.^14–17^ Furthermore, LTO in thin films is identified as a highly resistant candidate for ion-beam irradiation.^18^ The coexistence of various properties within a unique material seems exciting when many functions are required for applications. The specific NTO oxide explored in this work crystallizes as a single-crystal in a perovskite-layered monoclinic structure with a *P*2_1_ space group: *a* = 7.68(2) Å, *b* = 5.48(2) Å, *c* = 13.02(3) Å and *β* = 98°28′(5).^19^ It also has a *T*_c_ of 1482 ± 5 °C, a spontaneous polarization (*P*_s_) of 9 μC cm^−2^, and a coercive field (*E*_c_) of 200 kV cm^−1^ at room temperature.^4,19^ Recently, we successfully grew perovskite-layered ferroelectric NTO thin films through both a sol–gel route and pulsed laser deposition (PLD) on SrTiO_3_ (STO) substrates.^8,9,20^ Furthermore, the introduction of insulating STO layers within ferroelectric layers to form artificial superlattices has been shown to be promising for modulating the physical properties of thin films.^21–24^ For example, improved ferroelectric properties have been reported for BaTiO_3_ (BTO)/STO superlattices,^25–27^ while for BiFeO_3_ (BFO)/STO superlattices, a reduced leakage current was observed in the symmetric superlattices.^28–30^

Herein, we report the growth of (NTO_4_/STO_*n*_)_10_ (*n* = 4 and 8) superlattices on STO substrates using PLD combined with conventional reflection high energy electron diffraction (RHEED) system. In particular, the nanoscale electrical behavior and atomic arrangement were investigated to obtain a better understanding of the physical phenomena of these artificial structures. Epitaxial growth of the two superlattices was demonstrated using X-ray diffraction (XRD) measurements and RHEED pattern analysis, and was confirmed by scanning transmission electron microscopy (STEM) imaging. Conductivity variations were locally measured for the superlattices using conductive atomic force microscopy (c-AFM), with a higher leakage current obtained for the (NTO_4_/STO_4_)_10_ heterostructure. For this (NTO_4_/STO_4_)_10_ sample, no piezo-signal was obtained by piezoresponse force microscopy (PFM), while apparent piezoactivity was detected on the (NTO_4_/STO_8_)_10_ superlattice that was not primarily due to a pure ferroelectric phenomenon. Such electrical behaviors were principally attributed to point defects in the atomic structure from the substitution of Sr^2+^ by Nd^3+^ in the STO layers, as evidenced by STEM, and the existence of oxygen vacancies.

## Experimental

(NTO_4_/STO_4_)_10_ and (NTO_4_/STO_8_)_10_ superlattices (74–96 nm thick) and NTO single layers were grown by PLD on (110)-oriented 1.4 at% Nb-doped STO substrates (crystal, Germany) using a KrF excimer laser (Lambda Physik LPX, 248 nm wavelength, pulse width 25 ns). The base pressure in the chamber was reduced to ∼10^−8^ mbar. The substrate temperature during deposition was maintained at 600 °C and the deposition atmosphere was a mixture of O_2_ with 12% ozone at a fixed pressure of 5 × 10^−4^ mbar. The laser fluency was adjusted to 5 J cm^−2^ and the repetition rate was between 1 and 3 Hz. After growth, films and superlattices were slowly cooled to room temperature over about 30 min under the deposition atmosphere. RHEED diagnostic was used to control the growth and monitor the surfaces and interface qualities of the NTO films and NTO/STO superlattices *in situ*.

For the NTO target, a highly dense ceramic, 2.54 cm in diameter and 5 mm thick, was prepared using a classical solid-state reaction pathway. Briefly, the NTO oxide was prepared from mixtures of dried oxides Nd_2_O_3_ and TiO_2_ in stoichiometric ratios and heated under air at 1100 °C for 12 h with intermediate grinding. The resulting powder was pressed into a disc and sintered at 1250 °C for 12 h. The XRD pattern confirmed the monoclinic cell characteristics of NTO with *a* = 7.6745(4) Å, *b* = 5.4661(1) Å, *c* = 13.0048(9) Å, and *β* = 98.4394(7)°. Further details regarding the synthesis parameters are detailed elsewhere.^9^ For the STO target, commercial polycrystalline STO (disk diameter, 2.54 cm; *a*_cubic_ = 3.905 Å) from crystal, Germany was used.

A standard two-axis *θ*–2*θ* diffractometer (*λ*_Cu-Kα1_ = 1.5406 Å) was used to investigate the structural quality of the films and superlattices. High-angle annular dark-field (HAADF) STEM images and energy dispersive spectroscopy (EDS) mapping were obtained using an FEI TITAN Themis 300 system.

Nanoscale electrical experiments were performed using a commercial AFM microscope (MFP-3D, Asylum Research, USA) under ambient conditions. Ferroelectric properties were probed with a Pt/Ir-coated silicon tip and stiff cantilevers (*k*, ∼3 N m^−1^) in dual AC resonance tracking (DART) PFM mode.^31^ Conductivity variations were measured through current mapping and *I*–*V* characteristics experiments using the c-AFM technique (ORCA module for MFP-3D instrument). A Ti/Ir-coated silicon-grounded tip and cantilevers with a stiffness of ∼2.5 N m^−1^ were used. All ORCA experiments were performed by applying a DC bias voltage of −5 V between the grounded tip and the conductive substrate on the same day without changing the probing tip.

## Results

**Fig. 1:**
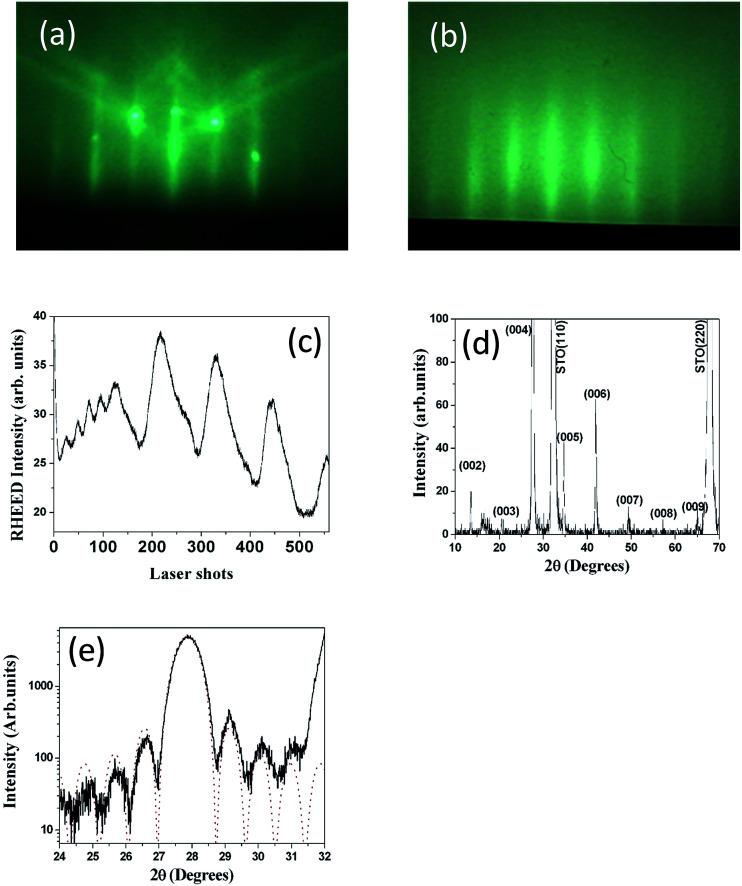
(a) SrTiO_3_ (STO) substrate RHEED pattern; (b) RHEED pattern observed after Nd_2_Ti_2_O_7_ (NTO) deposition; (c) RHEED intensity oscillations observed during NTO deposition; (d) *θ*–2*θ* XRD pattern of NTO film deposited onto (110)-oriented STO substrate; (e) XRD size effect interference fringes around the (004) peak of an eight-unit-cell-thick NTO film deposited on (110)-oriented STO substrate (dashed line corresponds to experimental data; dotted line corresponds to calculated data).

The structural properties of the NTO single layer were first characterized to confirm (00*l*)-epitaxial growth onto the (110)-oriented STO substrate, as previously reported when grown by PLD under standard conditions.^8,9^Fig. 1 shows RHEED patterns observed for the STO substrate before deposition (Fig. 1a) and NTO after deposition (Fig. 1b). When comparing these RHEED patterns, we assumed that the crystallographic relationships between NTO and STO were: [100]_NTO_//[001]_STO_, [010]_NTO_//[1̄10]_STO_, and (00*l*)_NTO_//(110)_STO_. As shown in Fig. 1c, the RHEED intensity oscillations observed during NTO deposition evidenced high-quality layer-by-layer NTO 2D deposition. Two main distinct features were noted. At the start of deposition, four RHEED intensity oscillations were clearly observed, indicating a period of about 25 laser shots for each oscillation. These first four RHEED intensity oscillations were attributed to deposition of the four-perovskite NdTiO_3_ unit cells. After those first four RHEED intensity oscillations, the period changed, now corresponding to about 115 laser shots. This second feature was attributed to the periodicity produced by the deposition of Nd_2_Ti_2_O_7_ unit cells. Notably, the period in this case was expected to be longer than that corresponding to deposition of the four-perovskite NdTiO_3_ unit cells. Therefore, from the RHEED intensity oscillations, we estimated a deposition rate value of about 0.1 Å per laser shot for NTO. The *θ*–2*θ* XRD pattern recorded after deposition (Fig. 1d) confirmed the (00*l*)-orientation of the film with respect to the (110)-plane of the STO substrate with *d*_00*l*_ = 12.88 Å, which led to *c* = 13.01 Å, as expected. In contrast, the full width at half maximum (FWHM) measured for the rocking curve recorded on the (004) reflection of NTO was 0.08°. This value was comparable with that obtained for the (110) STO substrate reflection, confirming the very low mosaic spread of the film and the high crystallographic quality of the deposited NTO. Furthermore, the NTO deposition rate estimated by the RHEED intensity oscillations was confirmed by the XRD size effect interference fringes around the (004) peak of an eight-unit-cell thick NTO film deposited on the (110)-oriented STO substrate, as shown in Fig. 1e.

**Fig. 2:**
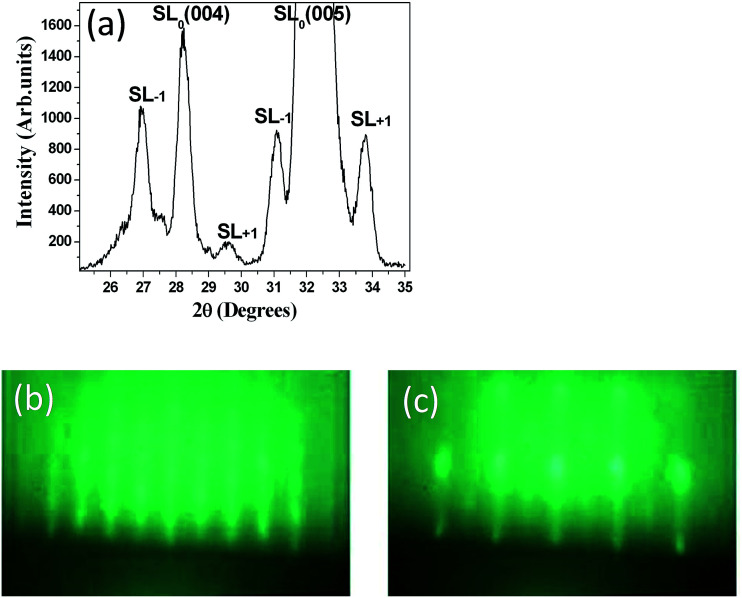
(a) XRD pattern obtained for (NTO_4_/STO_4_)_10_ superlattice. RHEED patterns recorded at (b) the end of deposition of the last NTO layer and (c) the last STO layer, for an (NTO)_4_/(STO_8_)_10_ superlattice.

Accounting for the deposition rate of NTO, various (NTO_4_/STO_*n*_)_10_ superlattices with different *n* values were deposited on similar substrates with different STO layer thicknesses. Khestanova *et al.* recently reported the fundamental role of the period in such complex artificial ferroelectric superlattices.^32^ The authors demonstrated that full-strain short-period superlattices showed a large polarization, which was not the case for longer multilayer periods, indicating that electrostatic boundary conditions were the main factor leading to the ferroelectric response at the expense of strain effects. From these results, our strategy was to fabricate superlattices with small *n* values, namely (NTO_4_/STO_4_)_10_ and (NTO_4_/STO_8_)_10_ (74 and 96 nm-thick, respectively). The XRD pattern obtained for the (NTO_4_/STO_4_)_10_ superlattice is shown in Fig. 2a. As expected, satellites peaks were observed around the two main peaks. The angular distance between the satellite peaks SL_−1_ and SL_+1_ around the SL_0_ for the (004) peak provided precise information on the film structure, allowing the lattice parameters of the superlattice unit cell to be evaluated. Through calculations accounting for the angular position of the satellite peaks, we confirmed the 4 × 4 periodicity of the synthesized (NTO_4_/STO_4_)_10_ superlattice. Similar results were obtained for the (NTO_4_/STO_8_)_10_ superlattice. Furthermore, the high quality of the diffraction peaks of these superlattices supported the epitaxial growth of these heterostructures.

During deposition of the (NTO_4_/STO_*n*_)_10_ superlattices, RHEED intensity oscillations were not observed. However the streaky RHEED patterns shown in Fig. 2b and c, and those recorded at the end of the deposition of both the NTO and STO layers, preserved the typical features of a quasi-2D deposition for each layer. Consequently, by considering the RHEED and XRD patterns, we found that good quality epitaxial NTO single layers of (NTO_4_/STO_4_)_10_ and (NTO_4_/STO_8_)_10_ superlattices were grown.

**Fig. 3:**
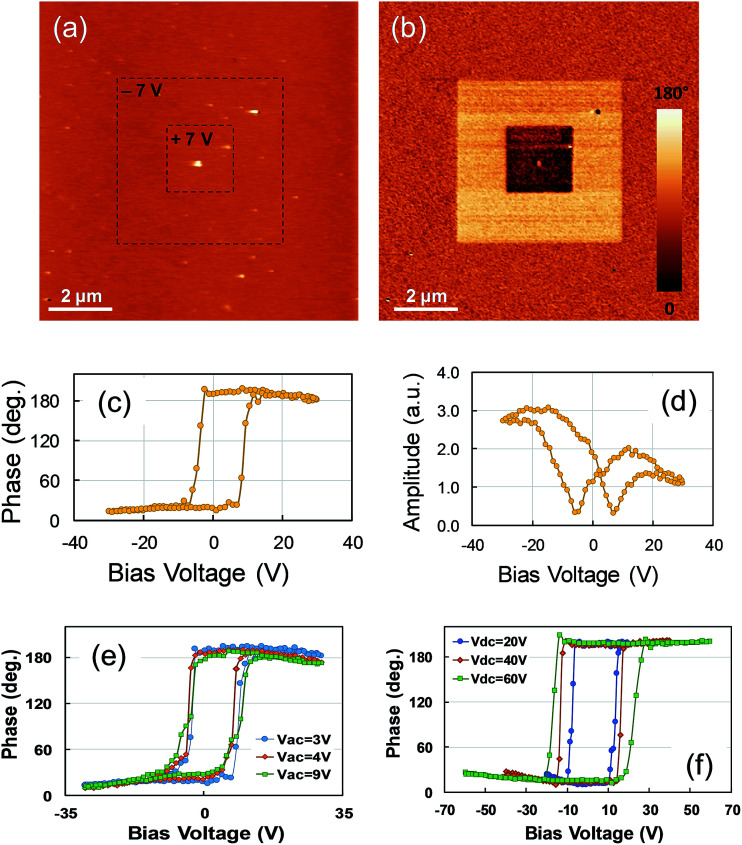
PFM experiments on the (NTO_4_/STO_8_)_10_ superlattice. (a) AFM topography and (b) out-of-plane phase PFM images recorded simultaneously. In the PFM images, one square region of 5 × 5 μm^2^ was poled (bright area), then a smaller square region of 2 × 2 μm^2^ was reverse poled (black area) by applying −7 V and +7 V, respectively. The scan area is 10 × 10 μm^2^ for both images. Remnant (c) in-phase and (d) in-amplitude piezoresponse loops. (e) In-phase piezoloops under different AC driving voltage and (f) in-amplitude piezoloops for different maximum DC voltage.

In a second step, characterization of the electrical properties of the two fabricated superlattices was conducted. Firstly, PFM experiments to determine the piezo/ferroelectric properties were performed on the (NTO_4_/STO_8_)_10_ superlattice, consisting of poling and switching experiments. Fig. 3a and b show the AFM morphology and corresponding out-of-plane PFM phase image, where two ‘artificial’ square-shaped domains of ‘upward’ (bright contrast, 5 × 5 μm^2^) and ‘downward’ (dark contrast, 2 × 2 μm^2^) polarization were locally written by applying DC voltages of −7 V and +7 V to the tip, respectively. Polarization switching seemed to be successful, as evidenced by both the well-defined squares and the uniform contrasts obtained after poling experiments, suggesting full switching of domains to one of the two c-oriented polarization states. Furthermore, no surface modification was observed on the corresponding topographic image, excluding surface electrochemical processes during poling experiments. To further address the switching behavior of ferroelectric domains, remnant piezoresponse loops on the superlattice were measured, as shown in Fig. 3c and d. The phase loop and butterfly-shape of the amplitude loop showed clear hysteretic behavior, suggesting the signature of local ferroelectricity in the superlattice, which was supported by applying the off-field recording method of the piezoloops to minimize the electrostatic effect.^33^

However, considering the complex nature of the physicochemical phenomena that can exist in such artificial superlattices (these ones include interlayer diffusion, oxygen vacancies, strains, discrete random and continuous layer thickness fluctuations at the interface)^34^ and in order to get a deeper insight on the ferroelectric-like behavior obtained, further PFM experiments were performed. In particular, PFM voltage spectroscopy measurements under different AC driving and maximum DC voltages were conducted. Fig. 3e shows the remnant in-phase piezoloops as a function of the driving voltage. As observed, the loops are insensitive when varying the magnitude of the *V*_AC_ probing voltage, especially when the *V*_AC_ was higher than the apparent coercive voltage (about 6 V). This specific behavior was a signature of a non-ferroelectric contribution to the observed PFM signal.^35–38^ From Fig. 3f, we noted a strong dependence of the remnant loops on the maximum DC voltage applied. Indeed, the opening of the loops continuously increased with increasing DC bias voltage, even once the apparent coercive voltage was reached. Again, this particular behavior cannot be only related to ferroelectric polarization.^39^ Consequently, these bias-dependent hysteresis measurements were unable to fully determine that the obtained PFM signals (poling and loops) were mainly due to the piezo/ferroelectric nature of the (NTO_4_/STO_8_)_10_ superlattice. Recently, much attention has been paid to such ‘false’ electromechanical PFM signals, which can be the signature of several phenomena, such as Joule heating, electrostriction, chemical dipoles, charge injection, field effects, Vegard strain, deposition, subsurface damage, surface damage, and vacancy ordering.^36–42^ Our results showed that the AFM-tip induced large voltages during PFM analysis, which could explain the hysteretic responses and opposite stable contrasts obtained on the (NTO_4_/STO_8_)_10_ structure by considering the existence of oxygen vacancies. Furthermore, as already demonstrated, a bistable distribution of such vacancies under electrical bias is responsible for the ferroelectric-like behavior in PFM imaging.^40,43^ Additionally, both the loop independence on the probing AC voltage with respect to the apparent coercive voltages and the area increase of the loops with increasing applied maximum voltage were related to an increase in the degree of ionic motion.^38,44^ Consequently, we considered ionic conduction and/or the motion of oxygen vacancies to be the main factors responsible for the poling contrasts and hysteretic responses obtained.

Similar PFM tests were undertaken on the (NTO_4_/STO_4_)_10_ superlattice, but no piezo-signal was obtained, regardless of the imaging method or spectroscopic mode of the DART PFM technique. This result was not expected considering that this short-period superlattice should have better ferroelectric properties, as shown by Khestanova *et al.*^32^

**Fig. 4:**
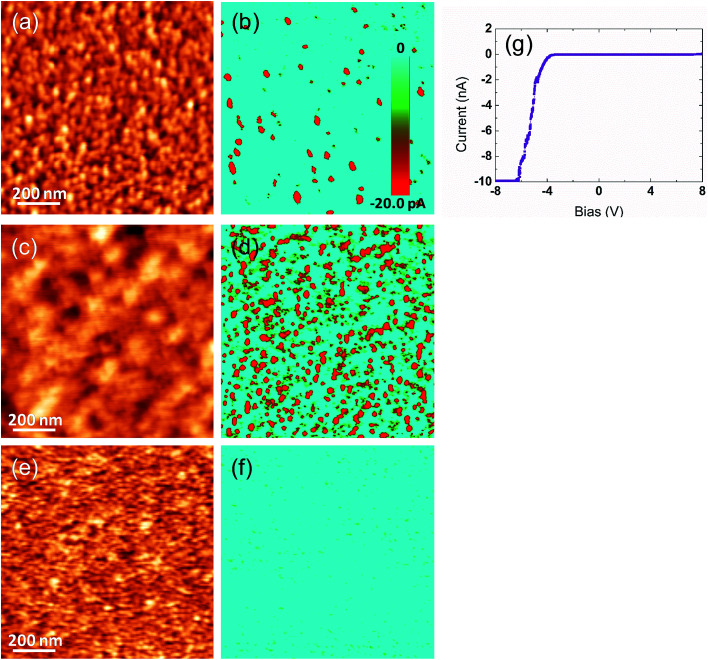
Local conductivity variations measured by c-AFM. Current mapping under −5 V DC bias measured over the surface of (b) the (NTO_4_/STO_8_)_10_ superlattice, (d) the (NTO_4_/STO_4_)_10_ superlattice, and (f) the NTO single layer. Red contrasts show higher local conductivity. Scale bar for the current is similar for all three images. AFM morphologies simultaneously recorded are shown in (a), (c), and (e). Scan area is 1 × 1 μm^2^. (g) Local *I*–*V* characteristics of the (NTO_4_/STO_8_)_10_ superlattice. At a bias voltage of −6.5 V, the detected current was higher than the detection limit.

At this point, c-AFM technique was used to locally probe the eventual leakage currents over the surface of the artificial superlattices. In particular, a leaky behavior could explain the absence of piezoactivity in the (NTO_4_/STO_4_)_10_ superlattice, and also help explain the ambiguous PFM responses observed on the (NTO_4_/STO_8_)_10_ superlattice. Furthermore, considering our assumption regarding the existing oxygen vacancies, such charged defects could lead to electrical conducting paths detectable by c-AFM. The current distribution was measured by applying a constant DC bias of −5 V between the grounded tip and the conductive substrate. The conductive Ti/Ir-coated AFM tip was used as a movable top electrode. Fig. 4b and d show current mapping with simultaneously recorded topography for the (NTO_4_/STO_8_)_10_ and (NTO_4_/STO_4_)_10_ superlattices, respectively. Variations in local conductivity were observed over the scanned regions, as evidenced by the red regions (‘hot spots’) in the blue background, where a higher conductivity of about −20 pA was detected. The distributions of the leakage current over the heterostructure surface were inhomogeneous, with no obvious correlation existing between the topography and the current image. This contrasted with results from other ferroelectric oxides, wherein both grains and grains boundaries represented a preferential electrical conducting channel.^45–49^ For positive bias, no local conductivity could be measured until +10 V, showing strong asymmetric leakage current characteristics. Local *I*–*V* curves were also recorded over the free film surface of the (NTO_4_/STO_8_)_10_ superlattice by sweeping a DC bias voltage, ranging from −8 and +8 V, between the conductive AFM tip and the heterostructure. The *I*–*V* characteristics presented in Fig. 4g show a diode-like behavior with a strong asymmetry in conduction behavior according to positive or negative voltages, which was consistent with the current map observed. Furthermore, no current was measured at a positive voltage, while significant conductivity was detected from a bias of around −4.5 V. From these results, a clear difference in detected current was observed between both superlattices, this was because the lateral density of the (NTO_4_/STO_4_)_10_ heterostructure significantly increased, despite the size of the ‘hot spots’ not changing, as shown in Fig. 4d. This improvement in the local conductivity could cause the PFM signal to be absent, and could be reasonably attributed to the smallest thickness of the STO slab (4 STO layers). However, as already mentioned, this contradicted with the recent work of Khestanova *et al.* on BTO/STO superlattices.^32^ To verify this assumption, the NTO single layer was also characterized by c-AFM under similar experimental conditions, and the obtained current map is shown on Fig. 4f. In this case, the detected current was found to be insignificant, as evidenced by the uniform blue contrast in the conduction pattern, which confirmed the insulator nature of the NTO film.

Therefore, the red contrasts observed on Fig. 4b and d suggested that the current passed through all STO layers, allowing for tunneling.^50^ To explain the drastic increase in conductivity when the number of STO layers was reduced to four in each slab of the superlattice, three main scenarios are proposed. The first was related to the existence of oxygen vacancies in the STO layers.^51^ In the case of LaNiO_3−*δ*_ (LNO) ultrathin films grown on STO substrate, Scherwitzl *et al.* hypothesized that the oxygen vacancy concentration might be larger near the interface LNO/STO.^52^ This would increase the relative fraction of nonstoichiometric material when the thickness of the film was smaller, explaining the observed metal-insulator transition when the film thickness was decreased.^52^ For our NTO/STO superlattices, we also believed the concentration of oxygen vacancies near the NTO/STO interface to be higher than that in the core of the STO layer. This should mean that the relative fraction of nonstoichiometric STO material, which contains mixed valence states of titanium (Ti^3+^/Ti^4+^), is higher in the (NTO_4_/STO_4_)_10_ superlattice than in the (NTO_4_/STO_8_)_10_ superlattice. Therefore, this phenomenon could favor conductivity in the (NTO_4_/STO_4_)_10_ superlattice, leading to an increase in electrical conducting channels, as observed in Fig. 4d. Furthermore, as mentioned above, such oxygen vacancies could explain the ambiguous PFM signals obtained for the (NTO_4_/STO_8_)_10_ structure. In the second scenario, the higher conductivity in (NTO_4_/STO_4_)_10_ superlattice compared with the (NTO_4_/STO_8_)_10_ superlattice can be explained by electronic miniband formation in such artificial structures. In their study on the theory of conductivity in superlattice minibands, Yang and Sarma showed that the mobility decreased when the period of the superlattice was increased.^53^ In our (NTO_4_/STO_*n*_)_10_ system, we have shown that the conductivity increased when *n* decreased. This result was in good agreement with the theoretical study^53^ and could explain the higher conductivity in the (NTO_4_/STO_4_)_10_ superlattice owing to a higher tunneling probability in this superlattice compared with the (NTO_4_/STO_8_)_10_ superlattice (whose periodicity was higher). However, the periods of our (NTO_4_/STO_4_)_10_ and (NTO_4_/STO_8_)_10_ as-grown superlattices were 74 Å and 96 Å, respectively which were larger than the 40–60 Å range for superlattice periods in the work of Yang and Sarma.^53^ Furthermore, as reported by the authors, the miniband width fell exponentially with increasing superlattice period. In contrast, considering that the thickness of the NTO barrier in both of our superlattices did not change, the increase in the number of STO layers from 4 to 8 should not decrease the conductivity as significantly, as shown in Fig. 4b and d.^54^ Therefore, we concluded that this second scenario was not the major cause of conductivity in our superlattices. The third scenario that might explain the higher conductivity in the (NTO_4_/STO_4_)_10_ superlattice is based on polar discontinuity existing across the NTO/STO heterointerfaces.^55^ As reported by Herranz *et al.* in the ideal ionic limit, the SrTiO_3_ perovskite structure stacks according to the sequence [O_2_]^4−^/[SrTiO]^4+^ along the [110] direction, which is along the growth direction of the NTO film.^56^ For the NTO film, along the [001] growth direction, the complex sequence was [O_2_]^4−^/[NdTiO]^5+^/[O_2_]^4−^/[TiO]^2+^/[Nd_2_O_4_]^2−^/[TiO]^2+^/[O_2_]^4−^/[NdTiO]^5+^.^17,57^ As a result, we assumed that the interfaces between STO and NTO should exhibit polar discontinuity. Furthermore, as reported by Ohtomo *et al.*,^55^ polarity discontinuities often arise in naturally layered oxide structures, such as ferroelectric titanates, including layered-perovskite phases constructed as A_*n*_B_*n*_O_3*n*+2_ for *n* = 2–6, and then the concerned Nd_2_Ti_2_O_7_ oxide corresponding to *n* = 4. However, considering that the number of STO/NTO interfaces was the same in the two (NTO_4_/STO_4_)_10_ and (NTO_4_/STO_8_)_10_ superlattices, we excluded this scenario to explain different maps measured on the superlattices. Consequently, we concluded that a more plausible scenario explaining the higher conductivity in the (NTO_4_/STO_4_)_10_ was related to the oxygen vacancies. Furthermore, cation interdiffusion/intermixing at heterointerfaces (Sr^2+^/Nd^3+^ in our superlattices) and epitaxial strain in the interfacial layers (STO/NTO in our heterostructures) and octahedral rotations (TiO_6_ in our case) through spatial gradient in the electronic structure are known to influence conduction.^56,58–60^

**Fig. 5:**
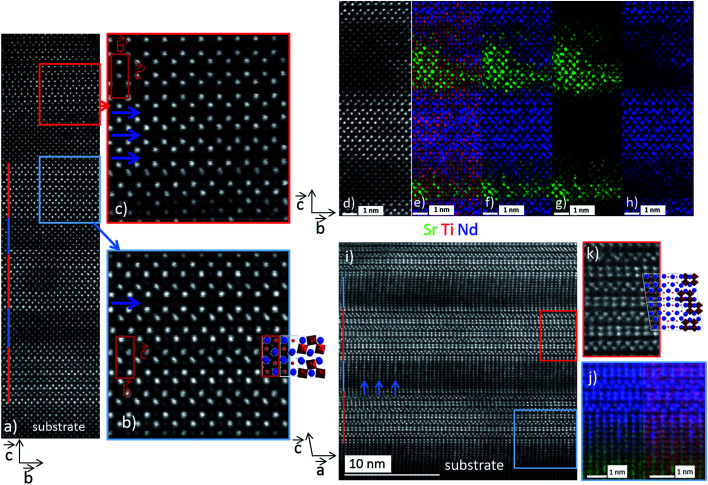
STEM-EDS analysis of the (NTO_4_/STO_4_)_10_ superlattice. (a) [100] HAADF image of the first four NTO layers. A regular stacking of 52.0 Å of NTO (red lines) and 22.1 Å (blue lines) of STO is observed. (b) Enlargement of the blue area in the third NTO layer showing a perfect stacking of the NTO, except at the level of the blue arrow, where a local defect is observed. (c) Enlargement of the red area in the fourth NTO layer where the previously defect is periodically repeated, leading to a new structure. (d) [100] HAADF image of the third NTO layer surrounded by two STO layers. EDS mapping of: (e) Sr (green) + Ti (red) + Nd (blue), (f) Sr (green) + Nd (blue), (g) Sr (green), and (h) Nd (blue). Significant Nd diffusion, which replaces Sr in the STO layers, is observed in this area. (i) [010] HAADF image of the first three layers. Red and blue lines indicate NTO and STO layers, respectively. Blue arrows show white lines in the STO layers, which were attributed according to (j) EDS cartography [Sr (green); Nd (blue); Ti (red)] of the substitution of Sr by Nd. (k) Enlargement of the red area showing the perfect [010] projection of the NTO structure.

**Fig. 6:**
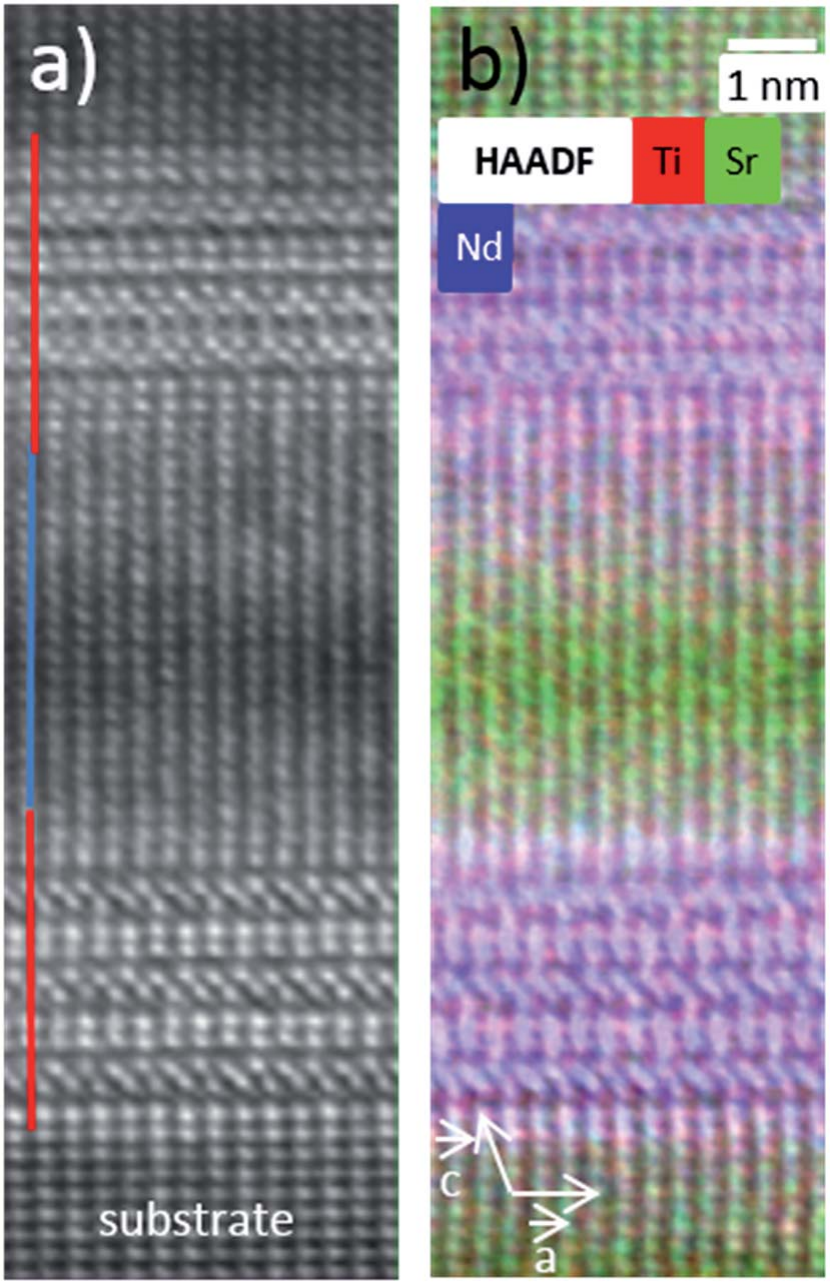
STEM-EDS analysis on the (NTO_4_/STO_8_)_10_ superlattice. (a) [010] HAADF image of the first two NTO layers. A regular stacking of 52.0 Å of NTO (red lines) and 44.2 Å (blue lines) of STO is observed. (b) EDS mapping showing a large diffusion of the Nd at the level of the STO layer [Sr (green); Nd (blue); Ti (red)].

**Fig. 7:**
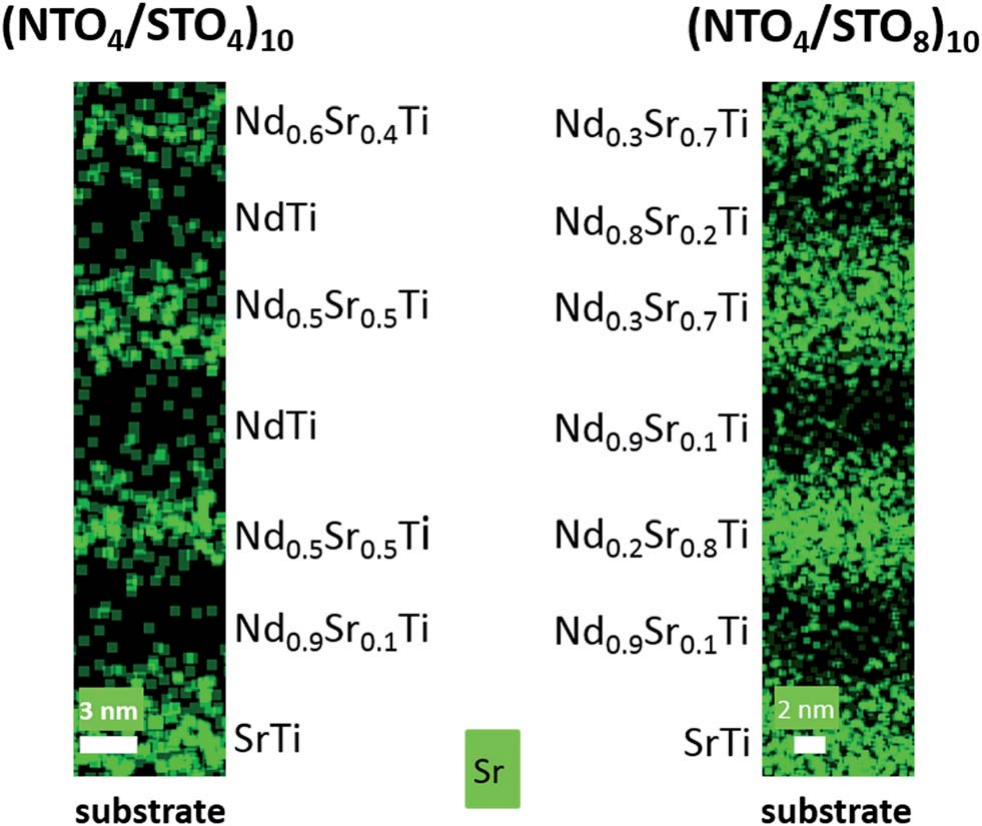
Comparison of the EDS composition of the first three NTO and STO layers in the (NTO_4_/STO_4_)_10_ and (NTO_4_/STO_8_)_10_ superlattices. Nd diffusion in the STO layer is greater in the (NTO_4_/STO_4_)_10_ superlattice. Low Sr diffusion is observed in the NTO layer of the (NTO_4_/STO_8_)_10_ superlattice.

At this stage, greater insight into the atomic structure and interface sharpness of the superlattices was required to explain the local electrical behavior observed above and validate the selected hypothesis. Therefore, we conducted a microstructural study using STEM on the cross-section prepared with a focused ion beam (FIB). Fig. 5 shows a HAADF-STEM image of the (NTO_4_/STO_4_)_10_ superlattice recorded along the [100] direction. The brightness of the dots on the image was proportional to *Z*^*n*^, where *Z* is the average atomic number of the atomic column. The column of Nd^3+^ ions appeared very bright (*Z*_Nd_ = 60), while the Ti^4+^ positions were seen as weak spots (*Z*_Ti_ = 22). As expected, perfect stacking of 4 NTO unit cells and 4 STO unit cells corresponding to 52.0 Å for NTO (4 × *c*_NTO_) and 22.1 Å for STO (4 × *a*_STO_√2) was observed (Fig. 5a). In the first three layers of NTO, the projection of the [100] structure was perfectly superimposed, except at the level of the blue arrow, where a local defect was detected (Fig. 5b). In the fourth and subsequent NTO layers, this local defect was generalized and periodically repeated, leading to a new structure (Fig. 5c). This could probably be attributed to the gamma phase often detected in NTO thin films and considered a polymorph of NTO.^61^ Structural characterization of this phase has yet to be established and will be explored in future work with the help of HAADF-STEM images, electronic precession crystallography, and electron energy loss spectroscopy (EELS) studies. The diffusion of Nd into the STO layers was considerable, as shown by EDS mapping in the region of the third NTO layer (Fig. 5d–h). The substitution of Sr with Nd in the SrTiO_3_ structure was not surprising considering the ionic radii of each cation. The ionic radius of Sr^2+^ in the 12-fold coordinated sites was 1.44 Å, and 1.24 Å when the coordination number was eight. In contrast, in the Nd_2_Ti_2_O_7_ structure, Nd^3+^ had ionic radii of 1.11 Å and 1.27 Å for 8-fold and 12-fold coordinated sites, respectively.^62^ These values were similar, explaining the large diffusion of Nd^3+^ cations observed on the Sr^2+^ sites in the STO structure. Projection of the [010]_NTO_ structure confirmed the previous results (Fig. 5i–k). Ordering the substitution of Sr by Nd was sometimes observed (blue arrows in Fig. 5i). Concerning the (NTO_4_/STO_8_)_10_ superlattice, the STEM HAADF images of the cross-section performed in the [010] direction of the first two NTO layers (Fig. 6a) also confirmed the stacking of four layers of NTO and eight layers of STO. As for (NTO_4_/STO_4_)_10_, the same new structure (as described in Fig. 5) appeared after the third NTO layer. EDS mapping (Fig. 6b) also showed the diffusion of Nd into the STO layers. However, this seemed less important than for the (NTO_4_/STO_4_)_10_ superlattice, with conservation of the structural integrity of Nd_2_Ti_2_O_7_ and SrTiO_3_ between these diffusion zones. To check the intensity of Nd diffusion at the level of STO layers, EDS compositions were estimated (Fig. 7). Nd was clearly more present in the STO layer of the (NTO_4_/STO_4_)_10_ superlattice. The ratio of Nd/Sr was higher in the STO layers of the (NTO_4_/STO_4_)_10_ superlattice than in the STO layers of the (NTO_4_/STO_8_)_10_ superlattice, which suggested that the diffusion and compositions were very different towards the boundary. In other words, Nd diffusion was more pronounced near the STO/NTO interfaces within the superlattices, as observed in Fig. 5f and 6b. From these results, we can directly link the local leakage currents observed by c-AFM to the diffusion phenomena for each superlattice. In particular, the higher cationic diffusion found for the thinner STO slab led to higher local conductivity, as observed in the current mapping (Fig. 4). This substitution of Sr^2+^ by Nd^3+^ in the SrTiO_3_ layer led to the mixed valence state of titanium (Ti^3+^/Ti^4+^), as evidenced by Joseph *et al.*^63^ Furthermore, when diffusion was stronger, in the case of the (NTO_4_/STO_4_)_10_ superlattice, the Ti^3+^/Ti^4+^ ratio increased and induced modification of the oxygen vacancies to preserve electroneutrality. Therefore, the higher Ti^3+^ content in the STO layers, combined with oxygen vacancies in the STO layers, would lead to higher conductivity in the (NTO_4_/STO_4_)_10_ superlattice. Furthermore, different mechanisms are known to explain the conductivity/leakage behavior in dielectric thin films and superlattices.^28–30,64–66^ Recently, Lu *et al.* showed the existence of highly conductive channels, ‘hot spots’, in ferroelectric SrTiO_3_ thin films emerging due to resonant tunneling through localized electronic states created by polar Ti_Sr_ antisite defects. The authors demonstrated that the defect-assisted tunneling mechanism, where conduction was dominated by ‘hopping’ through a defect state, played a decisive role in the local electrical properties of thin film SrTiO_3_ samples.^67^ Such conducting paths are very similar to those observed in our study, with identical behavior as a function of the STO thickness, which allowed the tunneling conduction mechanism to also explain the local conductivity found in our heterostructures. To carefully identify the conductivity mechanism existing in our superlattices, *I*–*V* curves and further AFM experiments will be considered in future work. Finally, the higher level of Nd diffusion associated with the larger electrical conductivity density might explain the absence of PFM signal in the (NTO_4_/STO_4_)_10_ superlattice, while lower diffusion corresponding to lower leakage current does not prevent the PFM signals being recorded, but was not mainly attributed to the ferroelectric phenomenon. Furthermore, this result was supported by the probable existence of oxygen vacancies and their migration beneath the probing AFM tip during bias application.

## Conclusion

Using a PLD technique with conventional RHEED, epitaxial NTO single layers and (NTO_4_/STO_*n*_)_10_ (*n* = 4 and 8) superlattices were grown on Nb-doped (110)-STO substrates. No piezoelectric signal was observed by PFM on the (NTO_4_/STO_4_)_10_ heterostructure, while ambiguous responses were measured on the (NTO_4_/STO_8_)_10_ heterostructure. The latter were typical of oxygen vacancy motion and/or ionic species migration under high electrical voltage. Electrical hot spots were locally detected on the surface of both superlattices, with a higher density when *n* was reduced to 4. EDS mapping showed Nd^3+^ diffusion in the STO layers, with a more pronounced effect observed for the (NTO_4_/STO_4_)_10_ superlattice. The existence of electrical conducting channels at the nanoscale in the superlattices was attributed to the presence of oxygen vacancies near the NTO/STO interfaces, along with the relative fraction of nonstoichiometric STO material, which increased when the thickness of each STO layer in the superlattice decreased. This high local current density prevented the measurement of PFM signals for the (NTO_4_/STO_4_)_10_ superlattice, while the PFM activity obtained for the (NTO_4_/STO_8_)_10_ superlattice could not be attributed to pure ferroelectric polarization behavior, as shown by bias-dependent hysteresis measurements. This study shows that precautions must be taken to carefully interpret local electromechanical signals obtained from classical imaging and spectroscopic modes of contact-resonance PFM. Furthermore, the interfacial structure properties were shown to be crucial for correctly elucidating the nanoscale electrical behavior in such artificial oxide superlattices.

## Conflicts of interest

The authors have no conflicts of interest to declare.

## Supplementary Material
